# Utilization of Magnetic Resonance Imaging by Comorbidity of Patients with Dementia

**DOI:** 10.3390/ijerph16234741

**Published:** 2019-11-27

**Authors:** Jihye Lim, Songhee Cheon

**Affiliations:** 1 Department of Healthcare Management, Youngsan University, Yangsan 626-790, Korea; limjiart@ysu.ac.kr; 2 Department of Physical Therapy, Youngsan University, Yangsan 626-790, Korea

**Keywords:** Dementia, MRI, CCI

## Abstract

Dementia produces major clinical and social problems that have catastrophic consequences for patients and their families. Dementia also complicates clinical care for other co-existing medical conditions. Magnetic Resonance Imaging (MRI) utilization is increasingly used for diagnostic purposes, such as early diagnosis of dementia and special examination of dementia. This study analyzed the utilization status and factors affecting use of MRI examination of patients with dementia using the Charlson Comorbidity Index (CCI). We used data from the Korean National Hospital Discharge In-depth Injury Survey (KNHDS) for three years, from 2013 to 2015, investigated by Korea Centers for Disease Control and Prevention (KCDC). The subjects of the study were 643 patients whose primary diagnosis code according to the International Classification of Disease (ICD) is F00–F03 (dementia in Alzheimer’s disease, vascular dementia, unspecified dementia, etc.). As independent variables, we used sex, age, type of insurance, the admission route, length of stay, result of treatment, number of hospital beds, and the hospital’s location. In this study, the independent variables affecting MRI examination of dementia patients were length of stay, hospital location, and CCI. The ratio of MRI examination of patients with dementia in which the CCI was 1, was significantly higher by 1.757 times than in cases where the CCI was 0. Hence, it can be used to provide basic data for formulating health care policy for dementia patients by studying their overall situation.

## 1. Introduction

The prevalence of dementia is increasing worldwide and is predicted to double every 20 years [1]. Dementia not only adversely affects the quality of life of patients but also leads to a costly burden of disease. As concern about dementia increases, interest in neuroimaging examination of patients whose cognitive function is decreasing is also rising. Brain Magnetic Resonance Imaging (MRI) examination has been used to detect lesions, such as brain atrophy, white matter lesion, cerebral hemorrhage, and latent cerebral infarction. However, MRI techniques have been developed recently for more than the detection of organic lesions. MRI utilization is increasingly used for diagnostic purposes, such as early diagnosis of dementia and special examination of dementia [2]. Dementia is the most representative senile disorder, and it requires a variety of medical services. According to earlier studies, most dementia patients have two or more comorbid conditions [3]. Comorbidity is defined as a clinical condition that exists at the time of the event that may affect the outcome of the study [4]. The Charlson Comorbidity Index (CCI) generates a total score that is weighted based on the presence of various conditions to account for different comorbidity and is commonly used in findings and mortality studies [5]. Comorbid conditions are more likely to affect the severity of dementia, as well as the socioeconomic burden and complexity of care [6]. According to a literature review, it was found that there is a difference in medical service use depending on comorbidities and the residence area of the dementia patient, and if patients with dementia have a higher CCI, they preferred large size hospitals with various care departments [7]. Since dementia patients have to consult with multiple specialists over extended time periods, the diagnostic workup of dementia is more difficult for rural patients [8]. As a result of the Korea Institute for Health and Social Affairs Research, the most common chronic disease for people with dementia is hypertension. The most common causes of death for patients with dementia were death due to cardiovascular disease, followed by respiratory disease and nervous system disease [9]. DeKosky [10] has suggested that diagnosis, treatment, and follow-up of dementia patients from the earliest possible stage will reduce health care costs, increase the quality of life for patients, and reduce family burden. Therefore, it is important that comorbid conditions are managed appropriately and there is effective diagnosis of dementia patients. MRI examination is useful not only for diagnosis of dementia but also for diagnosis of asymptomatic cerebral infarction. Asymptomatic cerebral infarction is known to exhibit a higher frequency in stroke and dementia patients; therefore, MRI examination of dementia patients can be extremely useful for the early detection of cerebral infarction [11,12]. Research on patients with dementia to analyze the effectiveness of an MRI diagnosis has recently been undertaken, but there is a lack of research on MRI diagnosis by comorbid condition of patients with dementia. To obtain more accurate data from MRI examination of patients with dementia, it is necessary to perform studies in which comorbid conditions are considered [13,14].

In this study, we analyzed the utilization status and factors affecting MRI examination of patients with dementia by using the CCI. Our results suggest essential data for the establishment of health care policy for improvement of quality medical care.

## 2. Subjects and Methods

We used data from the Korean National Hospital Discharge In-depth Injury Survey (KNHDS) for three years, from 2013 to 2015, investigated by Korea Centers for Disease Control and Prevention (KCDC). In February 2018, to acquire the data for the present study, the researchers went through the procedure for consent, including the application form for the use of raw materials and the pledge of information security through the injury Monitoring Business Homepage of the Center for Disease Control and Prevention (Statics Korea, Approval No. 117060). The Korean National Hospital Discharge In-depth Injury Survey (KNHDS) contains data collected from about 150 hospitals nationwide, each with more than 100 beds [15]. The survey items include gender, age, type of insurance, primary diagnosis, secondary diagnosis, admission route, length of stay, discharge form, etc.

The subjects of the study were 643 patients whose primary diagnosis code by the International Classification of Disease (ICD) was F00–F03 (dementia in Alzheimer’s disease, vascular dementia, unspecified dementia, etc.). Among the patients admitted to the hospital, we targeted patients whose primary diagnose was dementia. So, whether the diagnosis of dementia was given during the hospitalization or if the patients had pre-existing dementia is not clear. However, during the hospitalization period, the presence or absence of an MRI examinations was investigated, and the same patient may be hospitalized twice or more with dementia during the investigation period.

**Figure 1:**
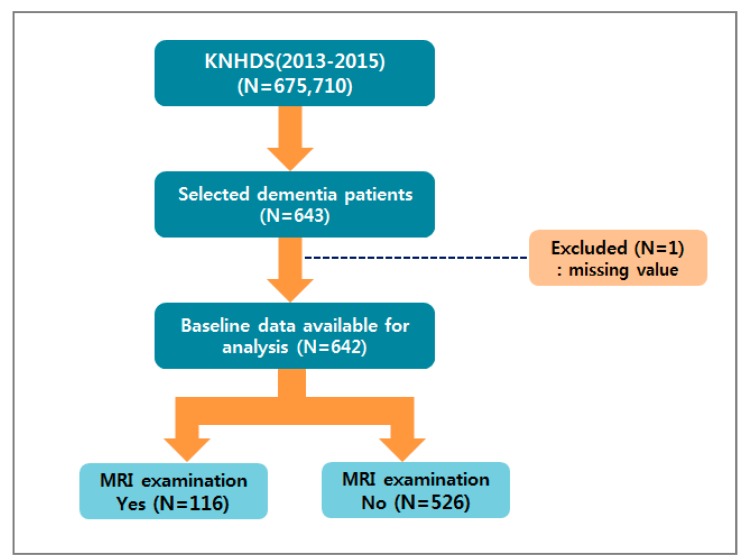
Flow chart for sample selection. KNHDS: The Korean National Hospital Discharge Survey.

The dependent variable was the presence or absence of MRI examinations, and the brain MRI code 88.91 was utilized according to the International Classification of Disease 9th revision Clinical Modification (ICD-9-CM) code (Figure 1). As independent variables, revealing socio-demographic characteristics, we used sex, age, and type of insurance. Type of insurance was defined as national health insurance, medicare, etc. The admission route, length of stay, result of treatment, number of hospital beds, and hospital location were used as independent variables, showing medical use characteristics. The length of stay was divided into 1–4 days, 5–9 days, 10–25 days, and 26 days or more, depending on the quartile. The result of treatment was divided into improved, not improved, diagnosis only, and death. The division of the hospital location considered the population number, the size of the city, the standard of the administrative area, etc. The hospital location was divided into Seoul, metropolitan, Gyeonggi, and other areas. Because Gyeonggi seemed to have similar characteristics to Seoul, it was divided into Gyeonggi and other areas. The Charlson Comorbidity Index (CCI) was used as a variable for evaluating the comorbidity of dementia patients. CCI is a method for providing a weighting from 1–6 for 19 diseases. We used classification scores of “0, 1, 2, and 3+” (Table 1) [16]. But, in this study, since patients with dementia as the primary diagnoses were included, dementia disease that correspond to a CCI score of “1” was excluded from giving a weighting.

Statistical analysis was conducted using the SPSS Statistics for Windows, version 24.0 (IBM, Armonk, NY, USA). Chi-squared analyses were conducted to ascertain the difference of MRI examination rate according to socio-demographic characteristics, CCI, and medical use characteristics of dementia patients. Logistic regression analysis was conducted to determine the factors that affect MRI examination of patients with dementia.

## 3. Results and Discussion

**Figure 2:**
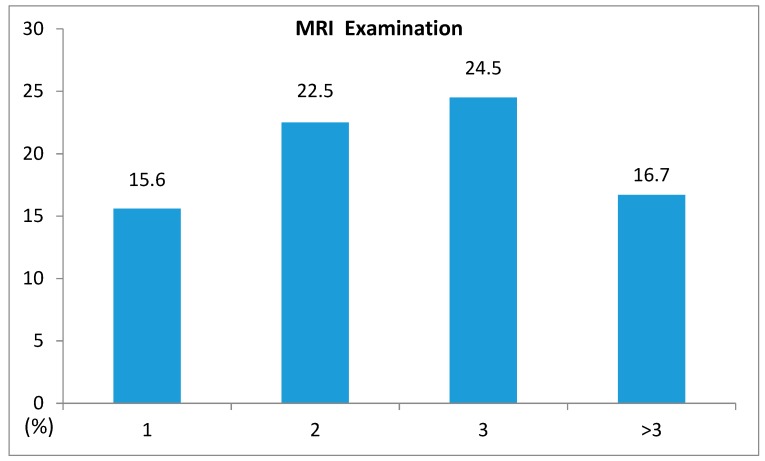
Distribution of Magnetic Resonance Imaging (MRI) examinations according to the Charlson Comorbidity Index.

Results of MRI examination rate according to the CCI of patients with dementia were 15.6% at CCI 0, 22.5% at CCI 1, 24.5% at CCI 2, and 16.7% at CCI 3 or more (Table 2, Figure 2). 

To examine differences in utilization of MRI for dementia patients by general characteristics, the Chi-squared test was conducted. The utilization of MRI according to dementia patients’ length of stay, result of treatment, number of hospital beds, and hospital location were significantly different (*p* < 0.05). The rates of MRI examination according to the length of stay were 26.5% at 5–9 days, 18.6% at 1–4 days, 18.3% at 10–25 days, and 9.0% at 26 days or longer. The rates of MRI examination according to the results of the treatment were at 28.6% in the case of diagnosis only, 20.0% at improved and 7.9% at not improved. The rates of MRI examination by hospital bed size were 28.4% at 500–999 beds, 14.4% at 100–299 beds, 12.8% at 1000 or more beds, and 9.1% at 300–499 beds. MRI examination rates according to location of hospital were the highest at 36.2% in the case of the Gyeonggi region (Table 3).

We conducted a logistic regression analysis to identify factors affecting MRI examination of patients with dementia. The independent variables were length of stay, CCI, and hospital location. The adjusted odds ratio (OR) of length of stay at 26 days or longer was 0.432 (95% CI = 0.209–0.896). In the case of 26 days or longer, compared with length of stay of 1–4 days, the ratio of MRI examination was significantly lower by a factor of 0.432. The ratio of MRI examination of patients with dementia in which the CCI was 1, was significantly higher by 1.757 times (95% CI = 1.070–2.884), compared to cases where CCI was 0. In the case of hospitals in the Gyeonngi area, compared with hospitals in the Seoul area, the ratio of MRI examination of patients with dementia was significantly higher by 2.645 times (95% CI = 1.278–5.474). These results suggest that the number of medical institutions in Seoul is higher than that in Gyeonggi area, but that the number of general hospitals and hospitals in the Gyeonggi area is larger than that in Seoul. Result of the likelihood ratio test was 536.081 (*p* < 0.05), therefore the null hypothesis of “regression coefficient equals zero” was rejected (Table 4).

Dementia is a broad term describing major neurological cognitive impairment due to brain disease or injury, which is most common in older people. Dementia produces major clinical and social problems that have catastrophic consequences for patients and their families. Therefore, early recognition and treatment of symptoms is essential to good practice. MRI is a necessary procedure for diagnosing dementia and essential for identifying the underlying cause of the dementia, whether it be degenerative, vascular, infectious, inflammatory, metabolic, or toxic [17,18]. 

The Comorbidity Index is used for different purposes in clinical, healthcare and research environments. In a clinical setting, indices can be useful in assessing a patient’s risk by creating a simple summary index [19]. CCI is widely used to study outcomes and mortality. In the present study, we investigated the utilization status and factors affecting MRI by CCI of dementia patients. 

Patients with dementia have usually many comorbidities. In this study, of all the patients with dementia, a CCI < 1 was seen in 88.0% of cases (565 cases). These results are due to the fact that minor diseases (hypertension, digestive order, arthritis, etc.) are not included in the CCI score. 

Our study showed that the ratio of MRI examination of dementia patients with a CCI 1 was significantly higher than for CCI 0 in the logistic regression analysis. With CCI 2 and 3+, the frequency of MRI was higher than seen with CCI 0, but the differences were not statistically significant. These results may be due to the small number of cases, and CCI weight and MRI frequency may be closely related as well.

Elderly patients may develop dementia that is associated with chronic diseases, mainly including chronic obstructive pulmonary disease, cerebrovascular accident, and heart failure [20]. MRI scans of patients with dementia may be useful for early detection of cerebral infarction [11]. In our previous research, the factors affecting MRI, which were critical for diagnosing cerebrovascular disease, included sex, age, type of insurance, admission route, length of stay, treatment result, location of hospital, and the number of hospital beds [21]. In the present study, the independent variables affecting MRI examination of dementia patients were length of stay, hospital location, and CCI. Both studies showed that the same factors were significant. Future research should focus on analyzing factors related to the efficient screening and diagnosis of high-risk patients with dementia.

This study has several limitations. Our study was a cross-sectional study, not a cohort study, and the subjects of the study, dementia patients, included both patients who were diagnosed with dementia before admission and those who were diagnosed with dementia. In addition, the data used in this study showed the presence of MRI during hospitalization and there was a limit to clarifying the causal relationship between diagnosis of dementia and MRI findings. However, it is considered to be meaningful data for the use of MRI according to the accompanying diseases of dementia patients, because it is based on the representative data collected from hospitals with over 100 beds in the Korea Centers for Disease Control and Prevention (KCDC).

## 4. Conclusions

In this study, there was a significant difference in the use of MRI among dementia patients according to the CCI. Except for the high-risk group, which is inappropriate for testing, the presence of comorbidity means that the diagnostic utility through MRI is higher. In addition, the higher utilization of MRI in a certain area (Gyeonggi) may reflect a large concentration of medical institutions in that area. In order to increase the utilization of MRI diagnosis and prediction through MRI, a separate program for early diagnosis of dementia that reflects these variables and appropriate distribution of medical resources by age, comorbidity, and region should be planned. Furthermore, the results of this study can be used as a basic data for programs for early diagnosis of dementia or for national health policy for the proper distribution of medical resources.

## Figures and Tables

**Table 1:** Assigned Charlson Comorbidity Index (CCI) according to the International Classification of Disease, 10th revision (ICD-10) codes.

Comorbidity	CCI Weight	ICD-10 Code
Myocardial infarction	1	I21.x, I22.x, I25.2
Congestive heart failure	1	I09.9, I11.0, I13.0, I42.0, I42.5–I42.9, I43.x, I50.x, P29.0
Peripheral vascular disease	1	I70.x, I71.x, I73.1, I73.8, I73.9, I77.1, I79.0, I79.2, K55.1, K55.8, K55.9, Z95.8, Z95.9
Cerebrovascular disease	1	G45.x, G46.x, H34.0, I60.x-I69.x
Chronic pulmonary disease	1	I27.8, I27.9, J40.x–J47.x, J60.x–J67.x, J68.4, J70.1, J70.3
Connective tissue disease	1	M05.x, M06.x, M31.5, M32.x–M34.x, M35.1, M35.3, M36.0
Ulcer disease	1	K25.x–K28.x
Mild liver disease	1	B18.x, K70.0–K70.3, K70.9, K71.3–K71.5, K71.7, K73.x, K74.x, K76.0, K76.2–K76.4, K76.8, K76.9, Z94.4
Diabetes	1	E10.0, E10.1, E10.6, E10.8, E10.9, E11.0, E11.1, E11.6, E11.8, E11.9, E12.0, E12.1, E12.6, E12.8, E12.9, E13.0, E13.1, E13.6, E13.8, E13.9, E14.0, E14.1, E14.6, E14.8, E14.9
Diabetes with end-organ damage	2	E10.2–E10.5, E10.7, E11.2–E11.5, E11.7, E12.2–E12.5, E12.7, E13.2–E13.5, E13.7, E14.2–E14.5, E14.7
Hemiplegia	2	G04.1, G11.4, G81.x, G82.x, G83.0–G83.4, G83.9
Moderate or severe renal disease	2	I12.0, I13.1, N03.2–N03.7, N05.2–N05.7, N18.x, N19.x, N25.0, Z49.0–Z49.2, Z94.0, Z99.2
Leukemia, Lymphoma, any tumor	2	C00.x–C26.x, C30.x–C34.x, C37.x–C41.x, C43.x, C45.x–C58.x, C60.x–C76.x, C81.x–C85.x, C88.x, C90.x–C97.x
Moderate or severe liver disease	3	I85.0, I85.9, I86.4, I98.2, K70.4, K71.1, K72.1, K72.9, K76.5, K76.6, K76.7
Metastatic solid tumor	6	C77.x–C80.x
Acquired immune deficiency syndrome	6	B20.x–B22.x, B24.x

**Table 2:** Distribution of MRI examinations according to the Charlson Comorbidity Index (CCI) of patients with dementia.

	CCI = O (*n* = 405)	CCI = 1 (*n* = 160)	CCI = 2 (*n* = 53)	CCI ≥ 3 (*n* = 24)	Total (*n* = 642)
MRI Yes *n* (%)	63 (15.6)	36 (22.5)	13 (24.5)	4 (16.7)	116 (18.1)

**Table 3:** Distribution of Magnetic Resonance Imaging (MRI) examinations according to general characteristics.

Variables		MRI Examination	Total (*n* = 642)	*X* ^2^	*p-*Value
		Yes (*n* = 116)			
Sex	Male	42 (16.7)	252 (39.3)	0.551	0.458
	Female	74 (19.0)	390 (60.7)		
Age	<60	11 (27.5)	40 (6.2)	7.592	0.108
	60–69	13 (21.7)	60 (9.3)		
	70–79	50 (20.1)	249 (38.8)		
	80–89	39 (15.3)	255 (39.7)		
	≥90	3 (7.9)	38 (5.9)		
Insurance type	National health	97 (18.4)	527 (82.1)	2.782	0.249
	Medicare	16 (15.0)	107 (16.7)		
	Others	3 (37.5)	8 (1.2)		
Admission route	Emergency	23 (15.5)	148 (23.1)	0.830	0.362
	Ambulatory	93 (18.8)	494 (76.9)		
Length of stay	1–4	31 (18.6)	167 (26.0)	16.106	0.001 *
	5–9	41 (26.5)	155 (24.1)		
	10–25	30 (18.3)	164 (25.5)		
	≥26	14 (9.0)	156 (24.3)		
Result of treatment	Improved	109 (20.0)	545 (84.9)	12.218	0.007 *
	Not improved	5 (7.9)	63 (9.8)		
	Diagnosis only	2 (28.6)	7 (1.1)		
	Death	0 (0.0)	27 (4.2)		
Number of hospital beds	100–299	47 (14.4)	326 (50.8)	20.637	0.000 *
	300–499	4 (9.1)	44 (6.9)		
	500–999	55 (28.4)	194 (30.2)		
	≥1000	10 (12.8)	78 (12.1)		
Hospital location	Seoul	19 (14.3)	133 (30.6)	27.636	0.000 *
	Metropolitan	22 (11.5)	191 (43.9)		
	Gyeonggi	34 (36.2)	94 (21.6)		
	Others	41 (18.3)	224 (34.9)		

* *p* < 0.05 by using a Chi-squared test.

**Table 4:** Factors influencing on Magnetic Resonance Imaging (MRI) examination of patients with dementia.

Variables		OR	95% CI	*p-*Value
Sex	Male	1		
	Female	1.236	(0.781–1.956)	0.365
Age	<60	1		
	60–69	1.068	(0.383–2.977)	0.899
	70–79	0.733	(0.314–1.712)	0.472
	80–89	0.578	(0.243–1.373)	0.214
	≥90	0.323	(0.075–1.394)	0.130
Insurance type	National health	1		
	Medicare	1.068	(0.567–2.014)	0.838
	Others	1.668	(0.336–8.288)	0.532
Admission route	Emergency	1		
	Ambulatory	1.329	(0.772–2.289)	0.305
Length of stay	1–4	1		
	5–9	1.245	(0.705–2.198)	0.451
	10–25	0.683	(0.374–1.248)	0.214
	≥26	0.432	(0.209–0.896)	0.024 *
Result of treatment	Improved	1		
	Not improved	0.467	(0.174–1.255)	0.131
	Diagnosis only	1.236	(0.200–7.639)	0.819
	Death	0.000	(0.000)	0.998
CCI	0	1		
	1	1.757	(1.070–2.884)	0.026 *
	2	1.706	(0.796–3.656)	0.169
	≥3	1.343	(0.388–4.653)	0.642
Number of hospital beds	100–299	1		
	300–499	0.458	(0.149–1.407)	0.173
	500–999	1.537	(0.911–2.591)	0.107
	≥1000	0.754	(0.308–1.844)	0.536
Hospital location	Seoul	1		
	Metropolitan	0.932	(0.440–1.975)	0.853
	Gyeonggi	2.645	(1.278–5.474)	0.009 *
	Others	1.472	(0.722–3.001)	0.288
Model Chi-square (df.) −2 log likelihood Nagelkerke R square		70.520 (23) 536.081 (0.000) 0.170		

* *p* < 0.05 by using a logistic regression analysis.
